# Evaluating the Quality of Social Work Supervision in UK Children’s Services: Comparing Self-Report and Independent Observations

**DOI:** 10.1007/s10615-018-0680-7

**Published:** 2018-09-21

**Authors:** David Wilkins, Munira Khan, Lorna Stabler, Fiona Newlands, John Mcdonnell

**Affiliations:** 1 CASCADE, Cardiff University, Cardiff, UK; 2 The Tilda Goldberg Centre for Social Work and Social Care, University of Bedfordshire, Luton, UK; 3 Children’s Services, London Borough of Islington, London, UK

**Keywords:** Children and families, Observation, Simulation, Social work, Supervision

## Abstract

Understanding how different forms of supervision support good social work practice and improve outcomes for people who use services is nearly impossible without reliable and valid evaluative measures. Yet the question of how best to evaluate the quality of supervision in different contexts is a complicated and as-yet-unsolved challenge. In this study, we observed 12 social work supervisors in a simulated supervision session offering support and guidance to an actor playing the part of an inexperienced social worker facing a casework-related crisis. A team of researchers analyzed these sessions using a customized skills-based coding framework. In addition, 19 social workers completed a questionnaire about their supervision experiences as provided by the same 12 supervisors. According to the coding framework, the supervisors demonstrated relatively modest skill levels, and we found low correlations among different skills. In contrast, according to the questionnaire data, supervisors had relatively high skill levels, and we found high correlations among different skills. The findings imply that although self-report remains the simplest way to evaluate supervision quality, other approaches are possible and may provide a different perspective. However, developing a reliable independent measure of supervision quality remains a noteworthy challenge.

Supervision is widely considered an essential form of support for good social work practice. In the United Kingdom (UK), as elsewhere, social workers employed by the state in children’s services are required to have regular supervision (Tsui 2005). Reasonably good evidence supports the claim that good supervision helps improve worker-related outcomes, including self-efficacy (Lee et al. 2011), confidence (Cearley 2004), stress levels (Boyas and Wind 2010), and retention (Kadushin and Harkness 2014; Mor Barak et al. 2009). However, little evidence clearly shows that supervision makes a difference for workers’ practice quality or client-related outcomes. Authors of a recent systematic review in the UK concluded, “The evidence base for supervision is weak” (Carpenter et al. 2013, p. 1851). In addition, researchers have debated the definition of good supervision. Researchers have emphasized different parts or elements of the process, although most have agreed that a good supervisor–supervisee relationship is foundational (Voicu 2017; Noble and Irwin 2009). Beyond this, researchers may place more or less emphasis on the importance of different skills, for example, problem solving (Lambeth Council n.d., p. 27), collaboration (Falender and Shafranske 2013), and reflection (Clayton 2017).

The primary method used to evaluate the quality of supervision in many studies is some variety of self-report. As commonly defined in methods textbooks, self-report does not necessarily mean participants provide personal information directly; self-report includes any method involving asking participants about their feelings, views, attitudes, beliefs, and experiences (Lavrakas 2008).

Wheeler and Barkham (2014) selected a “core battery” of six self-report measures to evaluate supervision components (pp. 367–385). These self-report measures included experience, focus, and ability (Orlinsky et al. 2005); the supervisory alliance (Efstation et al. 1990); and identification of supervision issues (Olk and Friedlander 1992). Davys et al. (2017) found that in daily practice, self-report is the most common way of evaluating supervision, most often through “informal discussions” between supervisors and supervisees, although some respondents reported using rating scales, questionnaires, and checklists as well (p. 114). The benefits of self-report include easy administration, low cost, face validity, and easy replication (Jupp 2006). Yet researchers have noted the well-known limitations of self-report, particularly in relation to evaluation (Fan et al. 2006; Huizinga and Elliott 1986).

First, people find it hard to assess themselves or others accurately, reliably, and consistently in relation to specific characteristics or competencies (Gurbanov 2016). As criminal defense lawyers and prosecutors have long known, “eyewitness testimony is unreliable [because] human perception is sloppy and uneven” (Buckhout 1974, p. 171). Thus, unless researchers take steps to correct biases, self-report must be interpreted with caution. Second, although it is possible to use rating scales to obtain responses more nuanced than simple yes or no answers, respondents are liable to interpret these scales differently. For example, one respondent might rate his or her satisfaction with supervision at 6 out of 10, and another respondent with a similar experience might rate his or her satisfaction at 8 (Austin et al. 1998). Third, respondents may interpret not only the scale but also the questions or statements in different ways. This may not be problematic for concrete questions (e.g., “How often do you have supervision?”) but may be troublesome for abstract concepts (e.g., “To what extent does your supervisor promote reflection and analysis?”). Fourth, the use of self-report methods to evaluate quality and outcomes is further complicated when the same respondents are asked to provide more than one type of data, as often happens in supervision and worker-outcomes studies. Mor Barak et al. (2009) summarized the problem as follows:

A [key] limitation stems from the potential for mono-method bias…, which is a typical risk when study respondents are the source of information for both the predictor and the outcome variables… Because most studies are potentially subject to mono-method bias, there may be some inflation in the results. (p. 26)

One possible solution is to use self-report methods with different respondents to assess different variables. For example, Harkness (1995) asked supervisees to rate the quality of their supervision, and clients were asked to rate various aspects of engagement and outcomes. Using this approach, Harkness found that the supervision skills of empathy and problem solving were associated with client ratings of contentment and goal attainment, respectively (pp. 69–70).

Another option is to develop evaluative measures that do not rely on self-report or that can be combined with self-report to increase validity and reliability. Bogo and McKnight (2006) called for the development of reliable supervision measures to facilitate comparison among different approaches in different contexts. Some researchers have sought to apply such measures to simulations of social work practice (Bogo et al. 2011; Maxwell et al. 2016). In addition, observations of real practice have been used as part of social work qualifying programs (Domakin and Forrester 2017) and in evaluative research studies (Bostock et al. 2017). Observational methods, whether simulated or real, are likely to be more valuable when researchers use a reliable and valid coding framework. Such frameworks enable evaluations that are more meaningful, which in turn fosters robust examinations of the relationships among supervision and other variables (e.g., family satisfaction with the service).

## Methods

In this paper, we report the results of a compare-and-contrast study using self-report data from social workers who rated the quality of their supervision. In addition, we used observations of how the same supervisors behaved in a simulated supervision session with a professional actor (Wilkins and Jones 2018). The methodological stance is one of theory-oriented evaluation (Weiss 1998). We began by providing in-depth descriptions of practice and then developed theories to explain how different elements linked and produced outcomes (White 2009). In this paper, we evaluate what happened in one particular form of supervision, or at least in a simulation of it, with the intention that the findings will inform further studies of how supervision shapes practice and outcomes. The overall method is one of participatory action research, with a focus not simply on describing what happens but also on helping supervisors and social workers reflect on their current supervision practices and outcomes.

### Context

In the UK, government organizations known as *local authorities* (of which there are 152 in England) typically provide statutory social work services for children. Each authority employs a number of social workers and supervisors to provide services for children and their families. The primary aim of these services is to protect children from significant harm resulting from abuse and neglect. Services include family support and other interventions. Unlike in some countries, social workers in UK local authorities receive supervision most often if not exclusively from their line managers (Wilkins et al. 2017). Typically, social workers are organized into relatively small supervision groups of approximately six people, supervised by the line managers. For the purposes of this paper, we were interested in the managers’ skills *in their role as supervisors*; thus, to avoid international confusion, apart from this paragraph, we refer to these participants as *supervisors* rather than *managers* (although in practice they fulfill both roles).

Over the past 2 years, along with many colleagues, we have been engaged in a large-scale participatory action-research project in one statutory children’s service in central London. At times, the project has involved participants from other local authorities as well. The project as a whole was funded by the UK Department for Education (Luckock et al. 2017). The primary aims of the project were to improve the quality of social work practice, to improve the experiences of children and families, and to reduce the need for children to enter public care. As part of this project, social workers were routinely observed in practice and supervision and were offered follow-up mentoring and feedback sessions (Wilkins and Whittaker 2017). We coded observations of practice using an established skills framework (Whittaker et al. 2016). We are currently developing a similar framework for supervision. This framework, coproduced with both supervisors and supervisees, is evolving; later in the paper, we describe in detail the version used in this paper.

As part of this iterative process, we became curious about the relationship between what social workers thought about their own supervision quality and what we thought about their supervision quality after observing it. This led us to develop the following research questions:

1. Using a customized coding framework, can we reliably assess the skills used by UK children’s services supervisors in simulated supervision sessions?
2. Using a self-report questionnaire based on the same framework, how do social workers assess the quality of their own supervision?
3. How do results from the two methods compare?

### Study Design

**Fig. 1:**
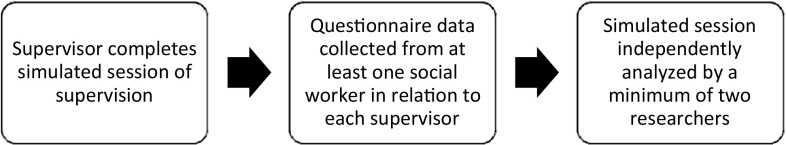
Outline of the three-stage data collection process

This study was undertaken in one outer London local authority with 12 supervisors and 19 social workers. In mid-2016, 12 supervisors took part in a simulated supervision session with an actor trained to play the part of an inexperienced social worker. In addition, we asked 54 social workers to complete a questionnaire about their experiences of being supervised by the group of supervisors who took part in the simulation. A group of five researchers with varying experiences and expertise in the field of child and family social work coded the audio recordings of the simulated sessions (Fig. 1).

### Ethics

The study received approval from the second author’s university ethics committee as part of the wider action-research project outlined previously. It was agreed that individual sessions would remain confidential unless serious concerns about malpractice emerged. This did not occur. Supervisors expressed their consent to take part in the simulations, and similarly, social workers consented in relation to the questionnaire.

### Data Collection

We used two methods of data collection—a simulated session of supervision, audio-recorded by the lead author, and a questionnaire completed by social workers. The simulation involved a professional actor playing the part of an inexperienced, newly qualified social worker asking for help in relation to a recent and concerning incident. The scenario occurred as follows: The worker, whose regular supervisor was on leave, received a telephone call from Elizabeth, mother to 5-month old Rees, with whom she had been working for approximately 3 months. Elizabeth reported to the worker that her ex-partner, Daniel, came to the family home last night and, under the influence of alcohol, attempted to take Rees away. When Elizabeth tried to stop him, he assaulted her. A neighbor called the police, who arrested Daniel but considered Rees safe enough to remain at home. The worker had arranged to visit Elizabeth but was unsure what to say and what other actions she might need to complete. The actor was advised to present as anxious and to express concern that Elizabeth could be concealing the true nature of her relationship with Daniel. Not all of these details were given to the supervisors beforehand—rather, the supervisors were briefed only that the social worker, sounding anxious, had asked to meet with them and that they had only 20 min before they needed to go to another meeting. Thus, the simulation was limited to a maximum of 20 min, although supervisors could have ended it sooner if desired. The lead author observed and audio-recorded each session for analysis by researchers blinded to the questionnaire data.

Social workers completed the questionnaire by hand on paper, separate from the administration of the simulation. The questionnaire consisted of two parts. In the first part, we asked social workers to report the frequency and length of a typical supervision session. The second part consisted of nine statements related to their supervisors’ supervision quality and the problems the supervision addressed. Respondents were asked to rate each statement on a 5-point Likert scale from *most agree* to *least agree*. Nineteen questionnaires were completed out of a possible 54, a response rate of 35%. This low response rate is typical. Baruch and Holton (2008) found from an analysis of 1607 studies that for questionnaires conducted with people who were members of organizations, the average response rate was 35.7% with a standard deviation of 18.8 (p. 1150). We collected at least one questionnaire for each supervisor, although for three of the supervisors, we collected two questionnaires each, and for two supervisors, we collected three questionnaires each.

### Data Analysis

At least two researchers coded each audio recording of simulated supervision using a customized supervision skills framework. Two researchers coded five of the simulations, three researchers coded four, and four researchers coded the remaining three. (Different numbers of researchers coded different numbers of recordings based on practical availability, rather than by intentional design—however, the lead author and at least one other researcher coded all the recordings.) The framework used in this study had three dimensions: clarity about risk and need, child focus, and support for practice. We used a 3-point Likert scale (1, 3, and 5) to give one score per dimension per recording. Inter-rater reliability was moderate; any disagreements were resolved through discussion among the relevant researchers (Table 1).

**Table 1:** IRR scores for researchers (percentage agreement and Krippendroff’s alpha)

Domain	Percentage agreement	Krippendroff’sα
Clarity about risk and need	68.50	.67
Child focus	80.00	.66
Support for practice	82.85	.71

We developed the coding framework used in this study as part of a larger action-research project. The framework, coproduced by researchers, supervisors, and supervisees, has been applied so far to more than 130 audio recordings of real supervision episodes from two different local authorities across a variety of different social work teams and services (including children in need/child protection, children in care, children leaving care, and fostering). The process has been iterative—we have revised and adapted the framework in relation to events in the sessions, based on feedback from supervisors and supervisees. Thus, the development of this framework is ongoing.

Researchers have proposed many definitions of good social work supervision, although we are not aware of any published measures that relate specifically to UK social work, other than Bostock et al.’s (2017) coding framework designed specifically for systemic group supervision. Many people would describe the characteristics of good supervision and good supervisors with some or all of the following phrases—communicating freely and reciprocally, encouraging the expression of authentic feeling, offering empathic understanding and acceptance, providing a problem-solving orientation based on consensus and cooperation and promoting a positive working alliance (Kadushin 1992).

In addition, in UK children’s services, ideas about good supervision may include considerations of children’s welfare (Reece 1996) as well as risk and need assessments (Skills for Care & Children’s Workforce Development Council 2007). Further, good supervision should support the quality of social work practice (Goulder 2013) without excluding good case management (Howe and Gray 2013, pp. 11–13).

These ideas have proven prescient for our work with the inner London authority. Through workshops and individual interviews with supervisors, we sought to develop a shared understanding of what constitutes good supervision in this particular context. The elements we agreed on through this process reflect many of those drawn from the literature (Table 2).

**Table 2:** The three core dimensions of good supervision to emerge from our action research project

Low		High
1	2	3
Clarity about risk and need		
• Limited or no mention of risks or needs • No attempt to prioritize risks or needs • Not linked to child/young person • Lack of curiosity, use of labels • Harm not discussed or discussed only generically • Not clear what needs to change • Risks/needs seen as static • No consideration of other perspectives, either professionals or family • Vague aims and goals	• Some references to risks or needs in relation to child/young person • Harm at least mentioned • What needs to happen/change is discussed mainly in terms of process • Some evidence of curiosity • Limited attempts to prioritise but may not be clear on what basis • One view of risks/needs dominates • Some attempt to individualise	• Related to child/young person, individually • Past and future harm discussed • Practice-led discussion about change • Extensive curiosity • Severity and change over time explicitly considered • Clarity about the bottom line and what needs to change • Risks/needs prioritised • Other views of risks/needs considered
Child focus		
• Child/young person absent from the discussion • Child/young person’s behavior not discussed • Adult needs dominate • Child/young person’s experiences not considered • Child/young person’s views not mentioned • Lack of knowledge	• Child/young person mentioned • Child/young person’s behavior mentioned • Adult needs more important • Child/young person’s experiences not considered • Some curiosity • Child/young person’s views mentioned • Some knowledge of daily life of the child/young person	• Behavior discussed and analyzed • Child/young person’s needs central • Child/young person’s experiences discussed extensively • Individualized discussion • Curiosity about the child/young person • Child/young person’s views important • Daily life of the child/young person known and understood
Support for practice		
• Focus is on process and management oversight • Deficit-based, in relation to the worker and the family • Lack of evaluation, limited curiosity about why things have been done/not been done, no attempt to learn from previous interventions • Advocates confrontation • Undermines confidence of the worker • Focus is on supervisor/agency needs • No help for worker	• Process is the priority, but practice is mentioned • Some strengths-based discussion, either about the worker or the family • No clear advocacy • Does not undermine confidence but does nothing to build it either • Some focus on the worker, but this is not the priority • Supervisor offers practical help • Some evidence of learning and evaluation but mostly case update information	• Practice is the priority/focus is on the worker and family • Clear advocacy for a strengths-based approach • Evaluative and open to learning from the past • Builds confidence, looking for ways to affirm the worker • Practical and critical support, combining practical help with developing worker skills • It matters whether supervision is helping the worker and the worker helping the family

The three dimensions in Table 2 formed the basis for our framework and questionnaire. We do not suggest these are the *only* important elements of good supervision; however, we agreed on these core dimensions through the process outlined previously. Again, in consultation with supervisors and social workers, we developed the core dimensions into a 3-point scale, with different descriptors for high-, moderate-, and low-quality examples.

The statements used in the questionnaire were designed to reflect the three dimensions of the coding framework. We used an average score from each set of three statements as an overall score for each of the three dimensions (Table 3).

**Table 3:** Questionnaire statements, organized by dimension

Clarity about risk and need
My supervision helps me think more clearly about risk
My supervision helps me think about immediate risk and longer-term risk
My supervision helps me think about how risks relate to the service user
Child focus
My supervision helps me think about how problems in the family might be affecting the child
My supervision helps me think about things from the child’s perspective
My supervision helps me focus on what is best for the child
Support for practice
My supervision helps me understand *why* I need to do things (not just what I need to do)
My supervision helps me understand *how* I need to do things (not just what I need to do)
My supervision helps ensure the quality of my practice

## Results

### Using a Customized Coding Framework, Can We Reliably Assess the Skills Used by UK Children’s Services Supervisors in Simulated Sessions of Supervision?

As a team of five researchers, we analyzed 12 simulated sessions of supervision in terms of three dimensions: clarity about risk and need, child focus, and support for practice. Across the 12 sessions, we achieved a moderate degree of inter-rater reliability (Table 1).

### Using a Self-Report Questionnaire Based on the Same Framework, How Do Social Workers Assess the Quality of Their Supervision Generally?

**Fig. 2:**
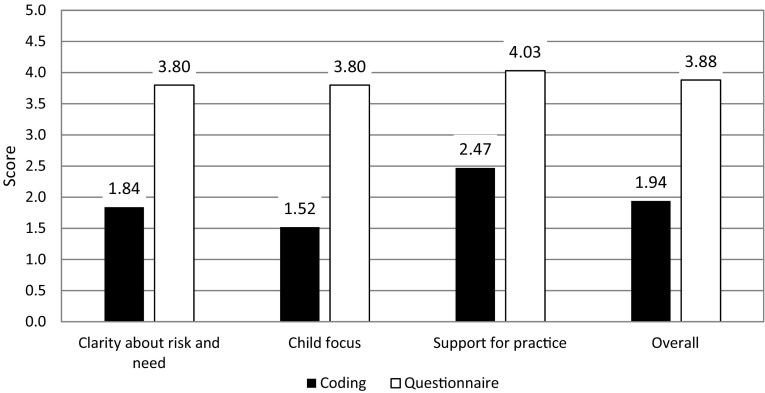
Comparison of coding scores (from researchers) with questionnaire data (from social workers)

Considering the same dimensions collected with the social work questionnaire, average scores were relatively high (Fig. 2). Based on the questionnaire data, we found strong correlations among the three dimensions (Table 4).

**Table 4:** Pearson correlations for the three dimensions based on social workers’ questionnaire results

		Clarity about risk and need	Child focus	Support for practice
Clarity about risk and need	Pearson correlation	1	.816**	.828**
	Sig. (2-tailed)		.000	.000
	*N*	19	19	19
Child focus	Pearson correlation	.816**	1	.785**
	Sig. (2-tailed)	.000		.000
	*N*	19	19	19
Support for practice	Pearson correlation	.828**	.785**	1
	Sig. (2-tailed)	.000	.000	
	*N*	19	19	19

**P < 0.01

### How do Results from the Two Methods Compare?

The scores provided by social workers indicate a skillful group of supervisors in relation to the three dimensions measured. However, the scores given by the research team indicate a less skillful group of supervisors (Fig. 2). Correlations among the dimensions as coded by researchers were weak. In addition, we found weak correlations between the scores given by researchers and the questionnaire data provided by social workers (Table 5).

**Table 5:** Pearson correlations between coding scores and questionnaire results

		Clarity about risk and need (coded)	Child focus (coded)	Support for practice (coded)	Clarity about risk and need (questionnaire)	Child focus (questionnaire)	Support for practice (questionnaire)
Clarity about risk and need (coded)	Pearson correlation	1	− .433	.228	.433	.400	.214
	Sig. (2-tailed)		.139	.453	.140	.176	.484
	*N*	12	12	12	19	19	19
Child focus (coded)	Pearson correlation	− .433	1	.158	− .130	.077	.000
	Sig. (2-tailed)	.139		.606	.673	.803	1.000
	*N*	12	12	12	19	19	19
Support for practice (coded)	Pearson correlation	.228	.158	1	− .174	.190	− .195
	Sig. (2-tailed)	.453	.606		.569	.534	.523
	*N*	12	12	12	19	19	19
Clarity about risk and need (questionnaire)	Pearson correlation	.433	− .130	− .174	1	.816**	.828**
	Sig. (2-tailed)	.140	.673	.569		.000	.000
	*N*	19	19	19	19	19	19
Child focus (questionnaire)	Pearson correlation	.400	.077	.190	.816**	1	.785**
	Sig. (2-tailed)	.176	.803	.534	.000		.000
	*N*	19	19	19	19	19	19
Support for practice (questionnaire)	Pearson correlation	.214	.000	− .195	.828**	.785**	1
	Sig. (2-tailed)	.484	1.000	.523	.000	.000	
	*N*	19	19	19	19	19	19

**P < 0.01

## Strengths and Limitations

The primary strength of the study is that it included direct observations of supervisors rather than relying solely on self-report. This remains a relatively rare approach in the study of supervision, albeit not a unique one. The primary limitation is that this study was based on a single simulated observation with an inexperienced worker (played by an actor) who the supervisors did not normally supervise. The impact of these features on the supervisors’ behavior is difficult to quantify; however, the nature of the scenario might indicate an action-oriented response rather than a reflective response. Nevertheless, social workers and supervisors are often encouraged to “reflect-in-action” as well as “reflect-on-action”, although some find this difficult at times (Ferguson 2018). In addition, many of the supervisors ended their sessions before the 20-min deadline. This could indicate that we timed the length of the simulation well, giving supervisors sufficient time to discuss everything to their satisfaction. Alternatively, it might indicate a level of discomfort and a desire for the experience to end sooner rather than later.

Other limitations include a lack of information about the characteristics of either the supervisors or the social workers. Further, we lacked knowledge about the questionnaire respondents, in particular, whether they differed significantly from social workers within the same supervision group who did not respond.

Finally, we acknowledge that the supervision framework we used is still in development. Although a similar version of the framework has been applied to actual supervision discussions, and the findings are reported elsewhere (Wilkins et al. 2018), researchers might consider this state of ongoing development a limitation. They might reasonably ask, why not wait until the framework is fully developed before publishing about it? We take a different view. We believe that publishing in relation to ideas still in development is a strength because publishing fosters criticism and feedback and thus potentiates future improvement. In any case, this paper is not principally about the framework; rather, it is about the difference between insider and outsider perspectives on supervision quality.

## Discussion

In discussing these results, the first thing to note is that this study forms part of an ongoing series of linked-but-separate projects focused on the nature and quality of social work supervision in UK children’s services. As such, we are not seeking to draw definitive conclusions from this study as a stand-alone project. Rather, we are interested in what it tells us about our approach to evaluating the quality of supervision and the implications of this approach more generally. However, before considering these general implications, we address three questions in relation to these results. First, why did our coding scores differ so much from the questionnaire results? Second, why did we find weak correlations among the coding scores across the individual dimensions? Third, why did we find strong correlations among the questionnaire scores across the individual dimensions?

### Why Did Our Coding Scores Differ So Much from the Questionnaire Results?

First, we discuss why our coding scores differed so much from the questionnaire results. One strong possible explanation is that we drew conclusions from a one-off observation, while the social workers provided feedback based on a far wider and richer range of experiences. As a research team, we listened to one simulated session of supervision, with no other knowledge about each supervisor. Domakin and Forrester (2017) found that making reliable judgments about practice skill required analyzing several observations, rather than just one. When completing the questionnaires, the social workers would have known far more about their supervisors and had experience of them in a much wider range of circumstances. Thus, the questionnaire results may not have reflected what happened in the observations (which, in any event, the social workers were not party to) but instead represented many weeks, months, or even years of experience.

In addition to collecting data in the outer London authority, we provided a workshop for the supervisors in this study, which took place after the simulations. The purpose of the workshop was to provide feedback to the supervisors regarding their collective performance and the anonymized questionnaire results. As part of the consent process before they completed the questionnaire, we informed the social workers they would be receiving feedback. However, although the questionnaires were anonymous, the social workers may have been reluctant to give negative feedback. Their supervision groups were relatively small (and the response rate modest), and it might have been easy for supervisors to decipher who completed each questionnaire. Individual social workers might have been wary of potentially disrupting their supervision relationships by giving challenging feedback and hence may have felt some pressure, consciously or unconsciously, to give positive feedback. This bias would not have influenced the research team, because we gave our feedback from a position of protected anonymity, and we had no ongoing relationship with the supervisors to protect.

This possibility resonates with the finding that the reality of an ongoing relationship between student social worker and practice assessor can complicate questions of objectivity and lead to inflated ratings of performance (Domakin and Forrester 2017). Finch and Taylor (2013) made similar arguments, suggesting that evaluating students is an emotional experience for many practice assessors. They concluded a strong possibility exists that at least some supervisors pass some social work students despite serious failings. Similarly, the social workers in our study might have felt an emotional response to being asked to rate the quality of their supervisors and responded accordingly in their feedback (see also Bogo et al. 2007).

Given the nature of the simulation, it would be understandable if the supervisors simply found it difficult and behaved differently than they might have in their daily work. The fact that they were encountering a stranger while being audio-recorded and assessed by an unknown team of researchers may have negatively affected their performances. They may have found themselves unable to adopt their usual approaches or demonstrate their typical skills. Perhaps some of the supervisors did not take the simulation seriously, given the pressures of their jobs. Participants would likely have believed it was more important to perform to the best of their abilities when real children and families were involved, whereas in the simulation, it did not really matter one way or the other. This attitude might account for the number of sessions that supervisors ended sooner than required, perhaps because they felt uncomfortable in the simulation or because they simply wanted to get back to their actual work. Thus, the coding scores given by the research team might be an accurate reflection of how the supervisors performed in the simulation, and the social workers’ questionnaire results might be an accurate reflection of how they performed more generally.

### Why Did We Find Weak Correlations Among the Coding Scores Across the Individual Dimensions?

Next, we consider why we found weak correlations among the coding scores across the individual dimensions of supervision skill. One explanation is that some supervisors may excel in some skill areas but not in others. For example, one supervisor might be skilled at assessing risk but less skilled in terms of focusing on the child. Another supervisor might be very good at focusing on the child but less able to support the quality of social workers’ practices. Such a conclusion would be analogous to believing that social workers can excel in some areas (e.g., engaging teenagers) while struggling in others (e.g., report writing).

Another possible explanation is that the simulation emphasized the demonstration of certain skills over others. For example, the social worker actor presented as anxious and unsure what to do next. This may have motivated a “support for practice” response among participants. In fact, we found that supervisors scored on average higher for this dimension than for the others.

It may also be the case that as a research team, we were more experienced at coding some dimensions, compared to others. This could have led us to give higher scores unintentionally for those skills. In a recent study, researchers found that the more experienced the assessors, the higher the scores they tended to give (O’Connor and Cheema 2018). This finding could show that rather than the supervisors behaving differently in relation to the different skills, the research team was simply more experienced at coding them.

### Why Did We Find Strong Correlations Among the Questionnaire Scores Across the Individual Dimensions?

Next, we question why we found strong correlations among the questionnaire scores across the individual dimensions. One consideration is that as researchers, we were rating the supervisors’ skills based on what we heard in the audio recordings, whereas the social workers would have been able to evaluate the relationship in a much more holistic way. For example, social workers with positive supervision relationships may have been consciously or unconsciously reluctant to give negative feedback about their supervisors, whereas social workers with more negative supervision relationships may have been similarly reluctant to give positive feedback. It is conceivable that the research team rated the supervisors’ specific skills (as described by our coding framework), whereas the social workers rated the overall relationship. If so, this would be an example of the “halo effect,” a form of cognitive bias whereby positive overall impressions influence the evaluation of more specific characteristics (Nisbett and Wilson 1977).

Finally, the coding framework we used is still in development and may not be a valid measure of supervisor skill (in addition to the limitations of using a simulation). This consideration may imply that our analysis of the audio recordings was not a meaningful indicator of supervision skill. In contrast, the social workers were likely to know their supervisors well; even if the questionnaire was not able to differentiate specific supervision skills, we find it hard to argue that the self-report feedback from the workers was not a valid reflection of how they felt about their supervisors and how they experienced their own supervision.

### What are the Implications of These Results for Wider Efforts to Evaluate the Quality of Social Work Supervision in UK Children’s Services?

Given these results, what are the implications for efforts to develop a reliable and valid framework for assessing the quality of supervision in the context of UK children’s services? First, albeit based on a small and nonrepresentative sample, the findings from our self-report questionnaire indicate that social workers tended to rate their supervisors either very highly or very poorly—there was no apparent middle ground. This finding implies that self-report, by itself, may lack nuance and sophistication, making it difficult to identify differences in quality and experience.

Second, our results indicate (if nothing else) that observing what happens during supervision may provide a different, rather than a complementary, perspective to self-report. This finding may be an unhelpful complication, or it may be a useful point of triangulation.

A third implication, and one that came up often in our discussions of the audio recordings, is that the local authority in question—and we suspect many others besides—did not have an accepted and shared vision of the nature and purpose of good supervision. Although researchers have done much in the UK in recent years to develop and implement frameworks for social work practice, less effort has focused on what makes for great supervision. Yet without such agreement, it is challenging to produce a coding framework that both makes sense to those being observed and that can be readily applied to different scenarios and contexts. After all, if supervisors do not consider supervision primarily a mechanism to support practice (as in clinical supervision), how helpful is it to code their supervision as if they did? Further, to what extent is it possible to develop a detailed coding framework based on examples that may or may not incorporate such attempts? Our findings show that when evaluating supervision, we need clarity about what we are trying to measure and why, as well as a shared understanding of what good supervision is or should be within a given context. Hence, in developing ways of measuring supervision, we need to remain mindful of the need to ensure the frameworks we use can be implemented reliably and that they measure elements that matter for practitioners and supervisors and ultimately for children and families.

## Conclusion

It seems likely (and desirable) that supervisors seek regular feedback from their supervisees in relation to the quality and helpfulness of the supervision they provide. In practice, much of this feedback is collected in relatively ad hoc fashion through informal discussions (Davys et al. 2017). Finding useful ways to collect feedback that is more structured would be highly advantageous. In the UK, many social work service leaders organize an annual “health check” survey of employees (Wolverhampton People Directorate Adult Social Care 2017; Local Government Association 2014), seeking feedback on a range of issues, including job satisfaction, employment conditions, and the quality of supervision support. Our findings show that although asking supervisees about their experiences of supervision remains a valid approach, it is important to acknowledge that different forms of evaluation will produce different results. Thus, leaders should think about ways of triangulating these data rather than relying on one method alone. Although self-report feedback may offer useful insights into how supervisees experience supervision, it can also mask the complexity and nuance of actual supervision case discussion outcomes, supervisors’ supervision skills, and where applicable, fidelity to a particular model of supervision.
